# Safety and Efficacy of an Intravaginal Prebiotic Gel in the Prevention of Recurrent Bacterial Vaginosis: A Randomized Double-Blind Study

**DOI:** 10.1155/2012/147867

**Published:** 2012-12-18

**Authors:** Isabelle Coste, Philippe Judlin, Jean-Pierre Lepargneur, Sami Bou-Antoun

**Affiliations:** ^1^Alliospharma, 1 Allée des Ecureuils, 69380 Lissieu, France; ^2^Department of Obstetrics, Gynecology & Reproduction, Maternité Régionale Universitaire, 10 rue du Dr Heydenreich, 54042 Nancy, France; ^3^Novescia, 29 rue de la bienfaisance, 75008 Paris, France

## Abstract

*Objective*. This study was performed to evaluate the efficacy and safety of a prebiotic treatment in the balance recovery of the vaginal flora in subjects previously treated for bacterial vaginosis (BV). *Study Design*. A randomized trial was carried out on 42 subjects with an active prebiotic group compared to a placebo group. The main evaluation criterion was the quantification of the vaginal flora measured by the Nugent score. Secondary criteria included vaginal pH and BV recurrence. *Results*. After 8 days of treatment, all subjects who received the prebiotic had a normal Nugent score, whereas 33% of the subjects treated with placebo had an intermediate or positive Nugent score. After 16 days of application, a normal Nugent score was maintained in all subjects treated with the prebiotic, whereas in the placebo group 24% of the subjects still had an elevated Nugent score. Moreover, the maintenance of (or reversion to) a normal flora was associated with the maintenance of (or reversion to) physiological pH values. *Conclusions*. The intravaginal gel treatment improves the recovery of a normal vaginal flora after the treatment of a BV episode, which should warrant a reduction in the risk of further recurrences.

## 1. Introduction

Bacterial vaginosis (BV) is the most common cause of abnormal vaginal discharge in reproductive age women [1]. Microbiologically, BV is associated with marked changes in microbial flora, characterised by loss or reduction of *Lactobacilli* and the concurrent high concentration of numerous other bacterial species, mainly* Gardnerella vaginalis, Atopobium vaginae*, but also* anaerobes* such as *Prevotella spp.* and Mobiluncus *spp.* [2]. The discharge results in part from degradation of the normal vaginal mucin gel, which is efficiently performed by mucin-degrading enzymes produced by BV-associated bacteria [3]. The odor, usually described as “fishy,” is derived from volatilization of the amines produced by the metabolism of anaerobic bacteria that characterize this disorder [4]. BV is not an infectious condition per se, though it can be responsible for adverse outcomes in pregnant women including premature ruptures of membranes, premature deliveries, chorioamnionitis, postpartum endometritis, and postpartum infant complications [5]. Studies have shown that risk factors for bacterial vaginosis are use of intrauterine devices, new or multiple sex partners, use of vaginal douches, or low levels of estrogen, for example, during menopause or due to oral contraceptives [6–9]. Standard treatment for BV consists of oral or intravaginal antibiotics, which are associated with an approximately 80% cure rate [10], these therapies are systemic or topical metronidazole and clindamycin. Previous studies reported cure rates of 48 to 96% for both antibiotics, with recurrence rates of 49 to 66%, following 7 days of therapy [11–13]. The treatments, while effective in addressing the abnormal proliferation of anaerobes, do not automatically restore a normal flora characterized by a high concentration of *Lactobacilli*, and this facilitates relapses and recurrences. 

Probiotics are live microorganisms that, when administered in adequate amounts, confer a health benefit on the host. In contrast, prebiotics are nondigestible food ingredients that beneficially affect the host by selectively stimulating the growth and/or activity of one or a limited number of bacteria. The rationale for the use of probiotics is mainly based on their ability to remodel microbial communities and thereby promote growth and survival of commensal bacteria in favour over growth of pathogenic bacteria. Furthermore, they exert immune-modulatory functions, influence, and promote epithelial cell differentiation, proliferation, and intestinal barrier function *in vitro*. 

Prebiotics stimulate the growth of one or a limited number of the potentially health-promoting endogenous microorganisms, thus modulating the composition of the natural ecosystem [14]. Up to now, prebiotics have mainly been studied in the intestinal ecosystem [15, 16]. Among the carbohydrates that are qualified as prebiotics, fructooligosaccharides (FOS) and glucooligosaccharides (GOS) are of interest [17–19]. The use of prebiotics to specifically promote the growth of vaginal *Lactobacilli *has not been well elaborated yet. We demonstrated after *in vitro* studies that a new prebiotic gel with APP-14, containing specific prebiotics from GOS family, was able to feed 3 endogenous vaginal *Lactobacilli* strains showing probiotic properties (*L. crispatus, L. vaginalis, *and* L. jensenii*) without promoting the growth of pathogenic germs such *C. Albicans* ATCC 2091, *E. Coli* CIP 548T, and *G. Vaginalis* CIP 7074T [20]. Our preliminary* in vitro* results led us to evaluate this new compound as a new treatment strategy to provide those *Lactobacilli* a competitive advantage in the *in vivo* urogenital environment (data not shown).

We performed this randomized clinical trial to evaluate the efficacy and safety of prebiotic gel with APP-14 use on the balance recovery of the vaginal flora in subjects previously treated for BV.

## 2. Material and Methods

### 2.1. Criteria for Eligibility

This double-blind, parallel placebo-controlled, randomized clinical trial was performed on premenopausal, nonpregnant women aged 18 to 50 years, attending the Hadyai Hospital (Thailand) for symptomatic BV and for which they received an oral course of metronidazole for 7 days. Subjects who were pregnant or nursing, allergic to one of the component of the tested product, with HIV infection or immunocompromised condition, were excluded. Other exclusion criteria were the use of intravaginal or systemic treatment during the week preceding the inclusion (or during the study) liable to interfere with the study evaluation. The trial was conducted in accordance with the declaration of Helsinki. The protocol was reviewed and given a favourable opinion by the Ethics Committee of Joint Research Ethics Committee (JREC) in Bangkok on March 5, 2009. Written informed consent was obtained for all participants.

### 2.2. Protocol Design and Treatment Modalities

Before enrolment, diagnosis of BV was made by clinical exam and confirmed from a vaginal sample using the Nugent score [21] and by counting the pathogenic germs *Mycoplasma hominis*, *Ureaplasma urealyticum*, *Gardnerella *vaginalis, and *Candida albicans*; in addition, a pH measurement was done. After the verification of inclusion and exclusion criteria, the informed consent was obtained, and then oral course of metronidazole was prescribed for 7 days. Subjects were randomized into two groups. One group received the active APP-14 gel inside a small tube containing 7 mL of product (minimum deliverable dose 5 g) with the GOS-alpha prebiotic (6%, equivalent to a minimum of 300 mg of oligosaccharide) and the trifolium pratense extract (2%), whereas the placebo group received the placebo gel without the two active ingredients. For all subjects, intravaginal gel was self-administered once a day for 16 consecutive days.

At day 0, day 8, and day 16, subjects were examined and material for laboratory tests was obtained. The subjects also answered the subjective evaluation questionnaire to assess the sensations of discomfort, burning, vaginal itching, leucorrhoea or vaginal discharge, or unpleasant odor. The main evaluation criteria were the quantification of the vaginal flora measured by the Nugent score after 8 days of treatment. The Nugent Score is a Gram stain scoring system from vaginal discharge in order to diagnose bacterial vaginosis, assessing the number of large Gram-positive rods (*Lactobacillus* morphotypes), small Gram-variable rods (*Gardnerella vaginalis* morphotypes), and curved Gram-variable rods (*Mobiluncus spp.* morphotypes).

 This score is currently the “gold standard” for BV diagnosis in Europe. Moreover, and thanks to its low cost, this test has the advantage of being feasible in virtually all laboratories with a very high reproducibility. The score can range from 0 to 10; a score of 0–3 is considered as normal, from 4 to 6 is intermediate, and a score from 7 to 10 is consistent with BV. 

Secondary evaluation criteria included Nugent score at day 16, pathogenic germ counts, evaluation of gynaecological scoring of the trophicity of the vulva and of the vaginal mucous membrane, vaginal pH, and product tolerance. BV recurrence was assessed by telephone interviews at day 84; the study flowchart is described in Table 1.

### 2.3. Treatment Allocation and Blinding

Each subject included in the study was assigned with an increasing number from 1, according to her order of inclusion in the study, and for random treatment group. The subject was assigned to receive the product labelled with the same subject number.

The Investigator (or delegate) was responsible for allocating the correct study product to the correct subjects according to the randomization number for the subject. A study product was never reassigned to another subject. Both investigator and subject were blinded for treatment allocation. Breaking the blind (for a single subject), that is, knowledge of the group in which the subject was assigned, was considered only when it was deemed essential by the subject's physician for the subject's care. Any intentional or unintentional breaking of the blind was reported and explained at the end of the study, irrespective of the reason for its occurrence

### 2.4. Sample Size and Statistical Analysis

Since the study was the first to investigate the effect of a vaginal prebiotic gel on the vaginal microflora, no reliable exact sample size calculation was possible. Due to promising *in vitro* results with the new prebiotic gel with APP-14 [20], we concluded that at least 20 subjects per group have to be included in the study. The statistical methods used to analyze data were chosen to detect results difference according to the sample size. The recoded Nugent score was compared at each visit day (D0, D8, and D16) using the chi-square test, in case of disregarded underlying assumption, a Fisher exact test was applied instead. The analysis of the gynaecological scoring of the trophicity and the subjective evaluation was done to compare the tested product and placebo using a Mann-Whitney *U* test. All tests were two-tailed and considered significant when *P* < 0.05. The analysis of efficacy data was performed on the intention-to-treat (ITT) population. An intermediary analysis has been scheduled on the 30 first subjects. All statistical analyses were carried out using the SAS software version 9.1 and SPSS version 14.0.

## 3. Results

### 3.1. Demographics

**Figure 1:**
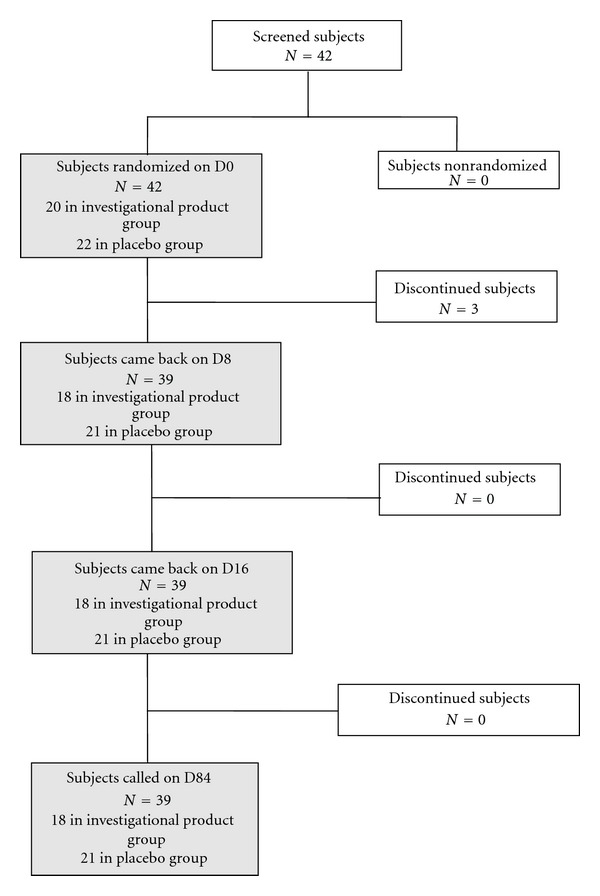
Subjects disposition in the study.

Between May 2009 and February 2010, a total of 42 subjects with a mean age of 33.64 years (min.–max., 21–50) were screened and found eligible for inclusion; 20 (47.6%) were randomized to receive the APP-14 gel while 22 (52.4%) subjects were randomly assigned to receive placebo; among them, 39 completed the study until the D84 followup (3 subjects were excluded because of major deviations from the protocol, two in the active group, and one in the placebo), the subject disposition in the study is shown in Figure 1. All subjects had a confirmed BV requiring antibiotic treatment at the preinclusion visit and received oral metronidazole during 7 days before treatment initiation (D0).

### 3.2. Nugent Score

**Figure 2:**
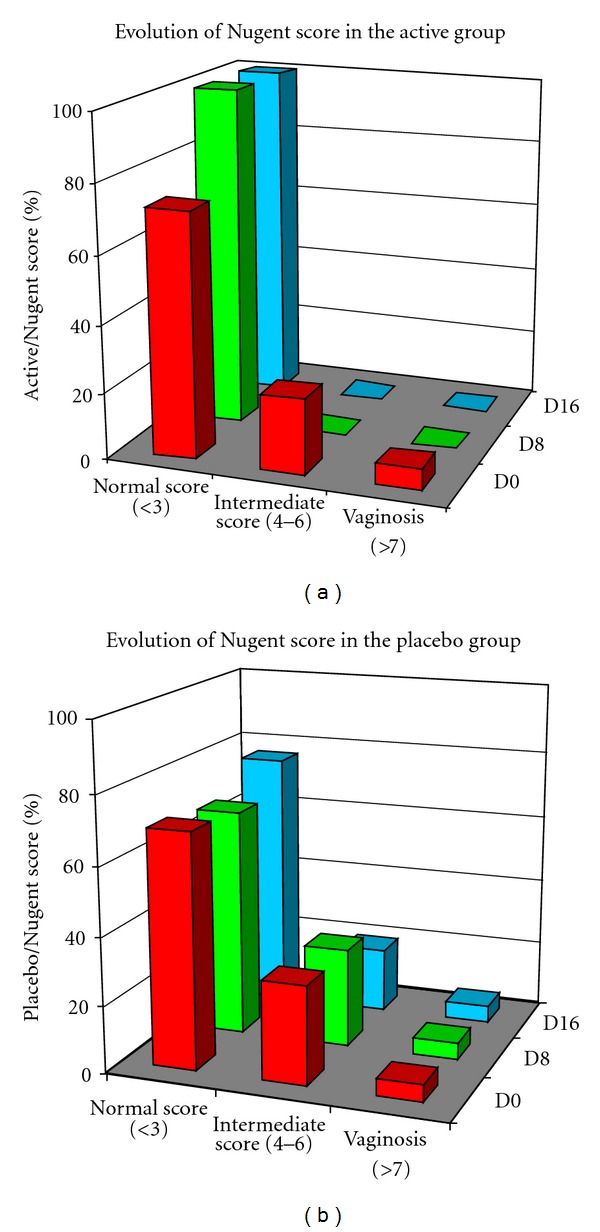
Schematic preview of Nugent evolution through visits.

After the antibiotic treatment at day 0, there was no significant statistical difference in terms of Nugent score between the active and placebo group (*P* = 0.296). After 8 days of treatment, all subjects who received the active treatment had a normal Nugent score, whereas 33% of subjects who received placebo had a Nugent score equal to or greater than an intermediate score. After 16 days of treatment, all subjects who received the active gel still showed normal Nugent scores, whereas 24% of subjects who received placebo still had a Nugent score equal to or greater than an intermediate score (Table 2). All data are statistically significant versus placebo. A schematic preview of Nugent score evolution through visits is shown in Figure 2.

### 3.3. Vaginal pH

The analysis of pH values was done in the light of the Nugent score. There was no significant statistical difference between the two groups in terms of pH values. In the group who received the active APP-14 gel, the stabilization of the Nugent score was associated in 62% of cases to a maintenance of a normal pH value and in 15% to a decrease in the pH value (i.e., from 5 to 4) while a pH increase was observed in 15% of cases (i.e., from 4 to 5). The reversion to a normal value of the Nugent score was correlated in 80% (4 subjects among 5) to a reversion to physiological pH values. In the placebo group, the stabilization of the Nugent score was associated in 71% of cases to the maintenance of the pH value and in 7% to a decrease in the pH value (i.e., from 6 to 4). The pH value increase was observed for 21% of the subjects. The reversion to a normal value of the Nugent score was observed in 43% of subjects, and it was correlated in 67% to a reversion to physiological pH values.

### 3.4. Vulva and Vaginal Mucous Membrane Trophicity

At baseline, the vaginal mucous membrane trophicity was generally good for all the subjects in both groups; this can be explained by the fact that study subjects are of young age. After 8 and 16 days, no significant change of vaginal mucous membrane trophicity score from baseline was observed between active and placebo groups.

### 3.5. Pathogenic Strain Germs Counting

Results of the pathogenic strains counting showed a difference between the active and the placebo group (not statistically significant because of the small sample size) in the number of *Candida albicans* and *Gardnerella vaginalis* isolated on D16. *Mycoplasma hominis* and *Ureaplasma urealyticum* were neither detected in the active nor placebo groups. *Gardnerella* morphotype and *Candida albicans* concerned one and maximum four subjects in the active and placebo groups, respectively (Table 3). 

### 3.6. Subjective Subject Evaluation

The functional signs reported by the subjects did not indicate relevant difference between the active and placebo groups. On D8 and D16, the statistical analysis of the data did not show significant difference between treatments in terms of “Product leakage” (resp., *P* = 0.8408 and 0.3767). No significant difference between the two arms was observed on D16 concerning the “Product ease of use” (*P* = 0.3092).

### 3.7. Recurrence of BV

On D84, subjects were called for questioning about potential BV recurrence. All the subjects present on D16 were called by the investigator (*n* = 39). Six subjects reported one new episode of BV: 2 (11%) in the active group and 4 (19%) in the placebo group. The two subjects who have received the active treatment and developed BV had initially poorly responded to antibiotic treatment and had a borderline Nugent Score (5 and 6) on D0. There was no BV recurrence in subjects who received the active and who had a normal Nugent score on D0. In the placebo group, 3 subjects out of 4 (75%) with BV recurrence had a normal Nugent score on D0 and D16.

### 3.8. Safety and Tolerance

At each visit, the investigator examined the subject and scored vaginal erythema, oedema, dryness, and leucorrhea (none, slight, moderate, severe, very severe). 

On D8 and D16, the investigator judged if the observed signs could be attributed with any relevance to the product. Additionally, subjects were also asked if they observed any physical signs after product application. The product was well tolerated and the tolerability was comparable between groups (active and placebo).

## 4. Comment

Our results showed that the prebiotic gel containing APP-14 was effective in restoring a normal vaginal flora (as assessed by the Nugent score) in all subjects, whereas 33% of subjects at D8 and 24% at D16 in the placebo group still had abnormal Nugent scores. The other evaluated parameters such as vaginal pH and cultures were not relevant and not significant in accordance with the Nugent score. The trophicity index was not assessable since all tested subjects were young and presented a normal vaginal trophicity at enrolment. Yet, this parameter will be a significant end-point in older users. Current recommended treatments of BV (in our study, Metronidazole by oral route for 7 days) seem to not fully restore a normal vaginal flora. The evaluation for recurrence performed at D84 was interesting indeed, though differences were not statistically significant (11% versus 19%) because the small size of the groups. This is in contrast to most of the currently available products (probiotics, pH enhancers) that claim properties and effects, which have not been supported by powerful scientific results. 

Prebiotics have been seldom studied in this field though they provide an interesting concept for improving vaginal flora quality. Previous data have shown controversial results in terms of efficacy on the use of probiotics, which consisted in different types and/or concentrations of *Lactobacilli* [22, 23]. Most of these probiotics have shown limited, if no effect in rebalancing the vaginal ecosystem because the strains commonly used are either gastrointestinal strains (e.g., *fermentum* or *casei*) or vaginal but with a nonsignificant presence (*rhamnosus* e.g., which represents less than 1% of the total microflora).

On the other hand, *in vitro* studies have shown that selected oligosaccharides significantly increased the growth of key species of *Lactobacilli* [20]. Our clinical study confirmed that this prebiotic topical compound was effective. Moreover, since there are limited variations among women in different ethnic groups in terms of the bacterial species found in the vagina, the results reported here should apply to women from different ethnical backgrounds [24]. The study was not able to check the clinical effects on trophicity since the enrolled women did not suffer from vaginal atrophy at inclusion. The scientific approach relying on topical prebiotics has never been tried before and one can observe it provides an original and interesting way to facilitate the growth of *Lactobacilli* in situations where they have been depleted. 

Finally, our results open a novel path of investigation into mechanisms of prebiotic function and, importantly, establish a proof of principle for the use of locally-administered prebiotics in bacterial vaginosis subjects. Therefore, the fast and reliable recovery of a normal vaginal ecosystem should warrant a reduction of the risk of further recurrences. 

## 5. Conclusion

An intravaginal prebiotic gel containing APP-14 effectively improves the recovery of a normal vaginal flora after the treatment of a bacterial vaginosis episode.

## Figures and Tables

**Table 1:** Study flowchart.

Schedule	Visit 1	Visit 2	Visit 3	Visit 4	Phone call
	Preinclusion visit				
Days		D0	D8	D16	D84

Antibiotic therapy prescription after clinical exam	■				
Enrolment	■	■			
Clinical examination by the gynaecologist (scoring)		■	■	■	
Vaginal sampling		■	■	■	
Vaginal pH measurement		■	■	■	
Subjective evaluation questionnaire on discomfort sensation and the medical devices		■	■	■	
Telephone interview inquiring about potential recurrence of the vaginosis					■

**Table 2:** Nugent score at the different visits.

		Treatment			
		Active		Placebo	
		*N* = 18	%	*N* = 21	%
Nugent scores at D0	0–3: Negative	13	72.22	14	66.66
	4–6: Intermediate	4	22.22	6	28.57
	>7: Indicative of BV	1	5.56	1	4.77
	Median, (min–max)	0 (0–7)		1 (0–7)	
	*P* value (active versus placebo)	0.296			

Nugent scores at D8	0–3: Negative	18	100.00	14	66.66
	4–6: Intermediate	0	0.00	6	28.57
	>7: Indicative of BV	0	0.00	1	4.77
	Median, (min–max)	0 (0–3)		1 (0–9)	
	*P* value (active versus placebo)	0.0478			

Nugent scores at D16	0–3: Negative	18	100.00	16	76.19
	4–6: Intermediate	0	0.00	4	19.00
	>7: Indicative of BV	0	0.00	1	4.81
	Median, (min-max)	0 (0–2)		0 (0–7)	
	*P* value (active versus placebo)	0.0157			

**Table 3:** Percentage of pathogenic germs in active and placebo groups.

		D0		D8		D16	
		Active	Placebo	Active	Placebo	Active	Placebo
Candida albicans	Absence	85	91	85	77	90	86
	Presence	5	5	5	18	0	9
Gardnerella morphotype	Absence	85	82	85	82	85	77
	Presence	5	14	5	14	5	18
Mycoplasma hominis	Absence	100	100	100	100	100	100
	Presence	0	0	0	0	0	0
Ureaplasma urealyticum	Absence	100	100	100	100	100	100
	Presence	0	0	0	0	0	0
